# Population structure of Cydia pomonella granulovirus isolates revealed by quantitative analysis of genetic variation

**DOI:** 10.1093/ve/veaa073

**Published:** 2020-09-29

**Authors:** Jiangbin Fan, Johannes A Jehle, Jörg T Wennmann

**Affiliations:** Julius Kühn Institute (JKI) - Federal Research Centre for Cultivated Plants, Institute for Biological Control, Heinrichstr. 243, 64287 Darmstadt, Germany

**Keywords:** population genetics, CpGV genotypes, consensus free, propagation quality, HCPC

## Abstract

Genetic diversity of viruses is driven by genomic mutations and selection through its host, resulting in differences in virulence as well as host responses. For baculoviruses, which are naturally occurring pathogens of insects and which are frequently sprayed on hundred thousands to millions of hectares as biocontrol agents of insect pests, the phenomenon of virus–host co-evolution is of particular scientific interest and economic importance because high virulence of baculovirus products is essential and emergence of host resistance needs to be avoided as much as possible. In the present study, the population structure of twenty isolates of the Cydia pomonella granulovirus (CpGV), including twelve isolates from different geographic origins and eight commercial formulations, were studied on the basis of next-generation sequencing data and by analyzing the distribution of single nucleotide polymorphisms (SNPs). An entirely consensus sequence-free quantitative SNP analysis was applied for the identification of 753 variant SNP sites being specific for single as well as groups of CpGV isolates. Based on the quantitative SNP analysis, homogenous, heterogenous as well as mixed isolates were identified and their proportions of genotypes were deciphered, revealing a high genetic diversity of CpGV isolates from around the world. Based on hierarchical clustering on principal components (HCPC), six distinct isolate/group clusters were identified, representing the proposed main phylogenetic lineages of CpGV but comprising full genome information from virus mixtures. The relative location of different isolates in HCPC reflected the proportion of variable compositions of different genotypes. The established methods provide novel analysis tools to decipher the molecular complexity of genotype mixtures in baculovirus isolates, thus depicting the population structure of baculovirus isolates in a more adequate form than consensus based analyses.

## 1. Introduction

Most of our knowledge of baculovirus functions is derived from laboratory studies of purified virus isolates containing a single genotype. To obtain such pure genotypes, isolates had to be purified, for example by plaque purification *in vitro* (Cooper 1962; Brown and Faulkner 1978; Durantel et al. 1998; Kariuki and McIntosh 1999; Harrison 2009; Gueli Alletti et al. 2018) or *in vivo* cloning (Winstanley and Crook 1993; Smith and Crook 1988; Luque et al. 2001). Naturally occurring baculoviruses, however, are often mixed populations of different genotypes, exhibiting genetic variation caused by insertions/deletions (indels) mutations (Crook et al. 1985; Thézé et al. 2014) and single nucleotide polymorphisms (SNPs) (Chateigner et al. 2015; Wennmann et al. 2017; Larem et al. 2019a). Such populations may have different biological characteristics than pure genotypes (Ferrelli et al. 2012; Gueli Alletti et al. 2017). With the advent of next-generation sequencing (NGS)-based deep sequencing, studying virus populations has been taken to a new level. Population structure can be described and analyzed, allowing insight into diversity as well as evolutionary constraints.

In the following, the population structure of Cydia pomonella granulovirus (CpGV) (genus *Betabaculovirus* of the family *Baculoviridae*,) was studied (Herniou et al. 2011). Its genome varies between 120.8 and 124.3 kbp in length and encodes 137 to 142 open reading frames (ORFs) (Wennmann et al. 2017). Due to the high virulence of CpGV against larval stages of the codling moth (CM, *Cydia pomonella* L.) and its wide application in biological control of this pest insect in pome fruit production, considerable efforts were undertaken to study this virus since it was first isolated from diseased CM larvae in Mexico in 1963 (Tanada and Leutenegger 1968; Lacey et al. 2008). This isolate, termed CpGV-M, was also the active ingredient in the first commercial CpGV product registered as a biocontrol agent (Huber 1998). As a consequence of these efforts, a large number of further naturally occurring isolates have been discovered from different geographic regions worldwide (Crook et al. 1985; Rezapanah et al. 2008; Gan et al. 2011; Arneodo et al. 2015; Fan et al. 2020a). Based on phylogenetic studies, CpGV isolates were grouped into seven phylogenetic lineages, termed genome groups A to G (Fan et al. 2020b). With the occurrence of CM populations resistant to CpGV products, scientific and economic interest in exploiting the genetic diversity of CpGV became even more significant (Asser-Kaiser et al. 2007; Sauer et al. 2017a,b). Nowadays several isolates obtained from natural environment and laboratory selections have been used in different commercial products worldwide, including the isolates from genome groups A, B, and E (Huber 1998; Vincent et al. 2007; Lacey et al. 2008; Zingg et al. 2011; Graillot et al. 2014; Gueli Alletti et al. 2017). To further characterize the genomic functions of CpGV and to identify the molecular nature of resistance-breaking CpGV isolates, genomes of different CpGV isolates were previously studied by Sanger sequencing and 454 pyrosequencing (Luque et al. 2001; Gebhardt et al. 2014; Wennmann et al. 2017). SNP pattern analysis was successfully applied to determine the identity and composition of natural and commercial CpGV isolates and to correlate their composition with different CpGV genotypes and their activity toward CpGV resistance (Gueli Alletti et al. 2017).

The picture of CpGV diversity and phylogeny was significantly extended when seven new Chinese isolates were characterized by NGS (Fan et al. 2020b): (1) in addition to previously defined genome groups A–E, two new phylogenetic lines (groups F and G) were found; (2) new SNP positions were identified; (3) based on the limited data sets used in previous studies, some recently identified group-specific SNPs were found to be not lineage specific anymore; (4) highly homogenous isolates and highly complex genotype mixtures could be identified in a single isolate.

As genotype mixtures are common in baculovirus field isolates (Xu et al. 2013; Thézé et al. 2014; Harrison et al. 2016; Larem et al. 2019a; Brito et al. 2018), a consensus sequence generated from an ultra-deep sequenced baculovirus isolate can only reflect the major frequency of every nucleotide base that was chosen from the assembly data (Chateigner et al. 2015), resulting in the loss of genomic information. In conventional phylogenetic analyses based on consensus sequences of baculovirus isolates, genetic compositions were very rarely taken into account. In the past, such limited methods were the consequence of low sequencing depths. For example, with the advantage of ultra-deep sequencing of Autographa californica multiple nucleopolyhedrovirus (AcMNPV) and the consequence of extremely high read depth, the vast majority (75%) of SNPs, however, with an extremely low frequency (0.01–0.27%), were detected, which requires the read depth of over 1,000 reads per nucleotide base in high-throughput sequencing (Chateigner et al. 2015; Loiseau et al. 2020). Previous studies of CpGV genomes were based on mainly Sanger sequencing or pyrosequencing techniques and more comprehensive NGS data were not available when the phylogenetic genome groups A to E were founded (Gebhardt et al. 2014; Wennmann et al. 2017). Because of the poor sequencing depth below 250 and the use of sequence consensus data for SNP analyses, no information was available on whether these isolates were also genotype mixtures and which SNPs were indeed isolate specific or genome group specific (Wennmann et al. 2017). This methodological limitation has been addressed in the current study by comparing new NGS data of the previously sequenced isolates CpGV-M, -I12, -S and -E2 (Gebhardt et al. 2014) as well as some new natural isolates (Fan et al. 2020b) and new laboratory selections. SNP detection of these newly and re-sequenced isolates were performed *de novo* in a consensus free approach, independently from previously identified SNPs (Wennmann et al. 2017). Variable numbers of 12 bp repetitive sequence motif in *pe38*, a resistance-overcoming marker of CM type I resistance, was screened using a read counting method (Fan et al. 2020b). Data were compiled and analyzed together with the NGS data of Chinese (Fan et al. 2020b) and commercial (Gueli Alletti et al. 2017) isolates extending the data set to 20 CpGV genomes. Analysis of SNP variation and frequency based on hierarchical clustering on principal components (HCPC) was applied on all variable SNP positions and alternative nucleotide frequencies. Principal components analysis (PCA) and hierarchical clustering (HC) are complementary methods, allowing for reduction in informational noise in the data and making clustering more robust (Husson et al. 2010). Such HCPC allows an improved representation of the diversity and composition of virus populations.

## 2. Materials and Methods

### 2.1 Geographic CpGV isolates and sequencing

Twenty different isolates of CpGV were included into this study, which were either field collected from different geographic locations or had been selected or formulated in a commercial context (Table 1). The geographic origins of the field collected samples were Mexico (CpGV-M), England (CpGV-E2), Canada (CpGV-S), Iran (CpGV-I12 and -I0X), and China (CpGV-ALE, -JQ, KS1, -KS2, -ZY, -ZY2, -WW). In this study, the isolates CpGV-E2 and -I12 were re-sequenced from virus samples that have previously been used for Sanger or 454 pyrosequencing (Gebhardt et al. 2014; Wennmann et al. 2017). CpGV-I0X was an un-characterized CpGV isolate originating from Iran (Rezapanah et al. 2008). The CpGV-M, -S and the seven Chinese isolates were ultra-deep sequenced in previous studies (Fan et al. 2020a,b; Wennmann et al. 2020) and their data were re-analyzed.

**Table 1.** Genomes of field origin and commercially formulated and selected isolates of CpGV that were sequenced and analyzed in this study.

CpGV isolate	Genome group	Reads (Phred quality score >30)	% reads mapped to CpGV-M	Mean read depth (±SD)	Country of origin (Company*)	Product name and additional information	Reference**
Field isolates							
M	A	3,650,570	99.4	3,995 ± 622	Mexico		4
E2	B	3,658,231	98.8	4,045 ± 725	England		1
I12	D	3,317,723	98.0	3,603 ± 593	Iran		1
I0X	D	3,141,095	96.9	3,406 ± 523	Iran		1
S	E	3,359,199	88.8	3,320 ± 614	Canada		4
WW	E	962,278	99.7	928 ± 204	China		2
JQ	F	1,022,825	94.4	950 ± 184	China		2
ZY	F	650,566	93.4	595 ± 121	China		2
ZY2	F	996,638	99.1	968 ± 195	China		2
ALE	G	1,181,528	92.5	1,076 ± 209	China		2
KS1	AD	1,134,546	96.3	1,057 ± 214	China		2
KS2	AD	879,222	90.5	776 ± 149	China		2
Commercial isolates							
R5	AB	3,102,552	15.7	540 ± 162	France (AL)	Carpovirusine EVO2^TM^	3
V003	A	1,573,038	98.7	1,301± 234	Switzerland (ABC)	MadexPlus^®^ (MPlus); selection from CpGV-M	1
0006P	AB	1,428,117	98.9	1,584 ± 438	Switzerland (ABC)	MadexMax^®^; batch no. 49	3
0006F	AB	3,485,678	96.5	3,734 ± 657	Switzerland (ABC)	MadexMax^®^; batch no. n.n	1
V15	BE	2,388,975	99.7	2,654 ± 448	Switzerland (ABC)	MadexTop^®^; batch no. 002	3
Commercially selected isolates							
0015	B	4,039,489	97.0	4,345 ± 818	Switzerland (ABC)	Formulation of CpGV-E2 (batch no. 11)	3
0017	–	2,110,218	99.6	2,361 ± 371	Switzerland (ABC)	selected in resistant CM larvae	1
V34	–	2,140,576	99.5	2,389 ± 404	Switzerland (ABC)	selected in resistant CM larvae	1

* AL, Arysta Lifescience; ABC, Andermatt Biocontrol.

** 1 = this publication, 2 = Fan et al. (2020b), 3 = Gueli Alletti et al. (2017), 4 = Wennmann et al. (2020). n.n, not available.

In addition to the field collected CpGV samples, commercially selected and formulated isolates were also included in this study (Table 1). Isolates with a commercial registration were CpGV-R5 (Carpovirusine EVO2^TM^; Arysta Lifescience, Noguères, France), as well as CpGV-V003 (MadexPlus^TM^), CpGV-0006P (MadexMax^TM^) and CpGV-V15 (MadexTop^TM^), all from Andermatt Biocontrol, Stahlermatten, Switzerland (Table 1). The sequencing data of CpGV-R5, -0006P and -V15 originated from Gueli Alletti et al. (2017) and was re-analyzed. The isolate CpGV-0006F described a different batch of CpGV-0006P and was first sequenced together with CpGV-V003 in this study. In addition to the commercially formulated isolates, samples of commercially selected isolates that were selected in resistance CM larvae and were considered as potential future biocontrol agents, were included in the population structure analysis of CpGV (Table 1). Besides CpGV-0015 (a formulation of CpGV-E2), -0017 and -V34 were sequenced (Table 1). For all CpGV isolates that were sequenced for the first time in this study, DNA was extracted from occlusion body suspensions using the standard protocol as previously described (Arends and Jehle 2002; Gueli Alletti et al. 2017). At least 100 ng of purified genomic DNA were sequenced using the Illumina NextSeq500^TM^ platform (StarSEQ GmbH, Mainz, Germany) with one to four million paired-end reads and 151 nucleotides in length (Table 1).

### 2.2 Processing of illumina reads

The processing of raw Illumina sequencing data of all 20 CpGV isolates was conducted in a highly standardized workflow on a JKI Galaxy server that applied the exact same parameters to each processed isolate. A detailed description of this workflow including additional information was published previously (Wennmann et al. 2020). At first all reads were adapter trimmed and quality filtered by using Trim Galore! v0.6.3 (Krueger 2019) with the following parameters: Phred score ≥30, minimal paired and unpaired read length of 50 and 51 nt, respectively. This resulted in the separation of reads of each isolate in three groups of paired, unpaired forward and unpaired reverse reads that were mapped subsequently against the common reference sequence CpGV-M (GenBank accession no. KM217575) with BWA-MEM v0.8.0 (Li 2013) using default parameters. At this step, it is important to mention that CpGV-M served as reference for the mapping of all 20 analyzed CpGV isolates. During the entire processing of reads, the affiliation of paired and unpaired reads to their corresponding isolate was ensured by the group identifier parameter as provided by the Galaxy server.

### 2.3 Detection of variable SNP position

The detection of SNPs was performed in a single step on all BWA-MEM output alignment files by using MPileup v2.1.1 (Li et al. 2009; Li 2011a,b). By using CpGV-M as the common reference sequence in the previous step, all detected variable positions were related to each other. Nonvariant sites were removed from the analysis by using BCFTools v1.0 (Li et al. 2009), which resulted in a data set of 753 variable SNP positions including the counts of the reference and the three possible alternative nucleotides.

### 2.4 Qualitative and quantitative SNP analysis

The entire set of 753 unique SNP positions was checked for all 20 CpGV isolates and with the four possible nucleotides in each position the entity of data points increased to 60,240. To reduce the data size and to remove non-relevant information, the data set was filtered by the bacsnp v0.1.0 package developed in R programming language (R v3.4.4 in RStudio v1.1.442) with the following parameters: only SNP positions at locations with an absolute total read depth >100 were considered; absolute alternative read counts should be higher than 10; the relative frequency (ƒ) of the reference or alternative nucleotide should exceed ƒ > 0.05. After filtering, the reference nucleotide and first alternative nucleotide counts explained approximately 100 per cent of the data, a finding that was described previously (Wennmann et al. 2020), and therefore the second and third alternative nucleotide were not included in this analysis (Fan et al. 2020b; Wennmann et al. 2020). Following these steps to increase the stringency of the data set, the specificities of SNP positions to certain CpGV isolates were determined under the following assumptions: (1) SNP positions were specific to one isolate only, when all other 19 CpGV isolates were missing alternative read counts in the same position and (2) SNP position were considered as group specific when two or more isolate were variable in these identical positions. The determination of SNP specificities was assigned by processing the filtered SNP data by the bacsnp v0.1.0 tool (Wennmann et al. 2020), which was established on the detection of SNPs by Mpileup (Li et al. 2009; Li 2011a,b). For the isolate-wide quantitative determination of mixtures, the average reference and alternative frequencies were calculated for all isolate-specific and group-specific SNP positions for each isolate separately.

### 2.5 ORF-associated SNP analysis

SNP density in every ORF was defined by the average SNP number in each ORF per kbp. On the basis of their annotation, ORFs were classified into biological regulation, DNA replication, metabolic process, structure protein, virus transcription and unknown function (Supplementary Table S2). Prior to comparison, the SNP density data were Tukey-transformed into normal distribution using rcompanion v2.0.10 R package (Mangiafico 2016). Significant differences were accessed by one-way analysis of variance (ANOVA) followed by Tukey's HSD (honestly significant difference) test for pairwise comparisons between ORF groups (R v3.4.4 in RStudio v1.1.442). Then the Tukey-transformed SNP density in all annotated ORFs was compared to that in ORFs with unknown function using Student's *t* test. This data matrix file was loaded into R software package.

### 2.6 HC on principal component

The variability of CpGV isolates represented by SNP positions and frequencies can be assessed by the distances between individuals using factorial analysis (PCA), HC and k-means clustering, of which three methods constitute HCPC. For the clustering of CpGV isolates, the filtered SNP frequency table with the entity of 753 SNP positions were used by applying the HCPC method as implemented in the FactoMineR v1.41 package (Lê et al. 2008; Husson et al. 2010) for R (R v3.4.4 in RStudio v1.1.442). The HCPC approach consisted of four steps: (1) factorial analysis (PCA), (2) HC, (3) cutting clustering tree, and (4) consolidation using k-means with the cluster centers. By default, the last factors were removed from analysis to lower the dimensions and to preserve the features in original data as much as possible, which makes the clustering more robust. Here, the first seven principal components or dimensions were selected for further clustering. PCA and HC are complementary approaches to cluster individuals together. Six clusters were set to correspond to the six CpGV genome groups A, B, D, E, F, and G (Gebhardt et al. 2014; Wennmann et al. 2017; Fan et al. 2020b) (Table 2). Hierarchical cluster tree and factor map were generated representing the lineages of highly homogenous, mixture and heterogenous CpGV isolates.

**Table 2.** Genetic composition of twenty field, commercial and commercially formulated isolates of CpGV.

	Median (%) (5–95%)																	
Genome group	A		B		D		F^#^		F		G		FG		BDEFG		DEFG	
CpGV isolate (No. of SNPs)	M (58)		E2 (68)		I12, I0X (24)		ZY2 (30)		ZY2, JQ (89)		ALE (21)		ALE, JQ (22)		WW, ZY2, KS2, KS1, ALE, JQ, ZY, S, I12, E2 (75)		WW, ZY2, KS2, KS1, ALE, JQ, ZY, S, I12 (45)	
M	100	(100–100)																
V003	100	(100–100)			0	(0–78)												
S	0	(0–1)													100	(100–100)	100	(93–100)
WW	0	(0–1)													100	(100–100)	100	(98–100)
KS1	69	(65–73)													29	(26–33)	30	(23–35)
KS2	86	(84–90)													14	(9–17)	14	(10–17)
ZY	77	(73–81)													22	(20–28)	23	(17–27)
ALE	94	(70–95)									80	(75–83)	80	(76–94)	6	(4–88)	6	(4–8)
ZY2	15	(0–95)					16	(13–18)	77	(14–81)					42	(4–100)	5	(4–84)
JQ	0	(0–84)							80	(2–84)			2	(1–3)	79	(15–100)	17	(13–100)
I12	56	(40–82)			2	(1–3)									46	(31–57)	42	(14–57)
I0X	93	(50–97)			35	(4–100)												
E2	20	(0–98)	84	(28–100)											67	(3–100)		
0015	27	(0–100)	81	(29–100)											60	(2–100)		
V15	7	(3–53)	42	(0–48)											93	(44–97)	49	(42–55)
0006F	26	(23–29)	0	(0–1)	0	(0–4)							0	(0–1)	75	(64–78)	74	(62–77)
0006P	32	(29–35)			0	(0–4)							0	(0–2)	68	(63–72)	68	(59–70)
R5	33	(24–64)			0	(0–8)									67	(29–76)	64	(30–72)
V34	38	(25–75)	0	(0–39)	0	(0–19)									67	(23–76)	28	(23–67)
0017	61	(0–98)	2	(0–72)	0	(0–67)									30	(9–99)	19	(5–71)

Median frequencies of SNP variants with correspondent 5–95% percentiles were measured based on their isolate specificity. Given are the previously named genome groups A, B, D, E, F, and G based on the phylogenetic reconstruction. F^#^, CpGV-ZY2 specific SNP contributing to genome group F. Groups labelled FG, BDEFG, and DEFG represent combined genome groups.

### 2.7 Counting of *pe38* repeat motif

Due of the experimentally validated role of *pe38* in breaking type I resistance (Gebhardt et al. 2014), the quantity differences of its 12 bp repeat motif of GACACAGTGGAT were analyzed according to the method described by Fan et al. (2020b). In brief, all quality-passed reads encompassing the entire *pe38* repeat region were counted when they fulfilled the following conditions: (1) they were defined by nonrepetitive flanking sequences of 12 bp upstream and 10 bp downstream of the 12 bp repeat motif and (2) both non-repetitive flanking sequences were present on a single read within the data set. From this entire set of reads covering the entire *pe38* repeat region the distance between the nonrepetitive flanking sequences was calculated for each read and isolate by the help of the ShortRead v1.40.0 (Morgan et al. 2009) and stringr v1.3.1 (Wickham 2018) package for R. The distances were divided by 12 resulting in a frequency distribution of the repeat length of *pe38* for each isolate.

## 3. Results

### 3.1 Illumina sequence data of twenty CpGV isolates

In total, NGS data of twenty CpGV isolates were compiled and analyzed (Table 1). On average, 91.9 per cent of 2,124,330 high-quality reads of each isolate were mapped against the CpGV-M reference genome sequence. The proportion of total reads mapped to CpGV-M was in the range of 88.8 to 99.7 per cent, except isolate CpGV-R5 that was contaminated with *C. pomonella* genome (Gueli Alletti et al. 2017). Mean read depth ranged from 540- to 4,345-fold (Table 1). These sequence data provided an adequate coverage for detection of genetic variants of all given isolates.

### 3.2 Variant detection and category

**Figure 1.**
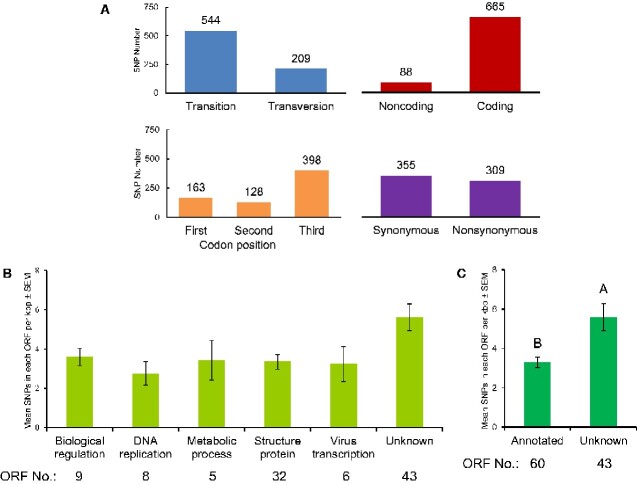
Classification of 753 SNPs identified in twenty CpGV genomes. (A) SNP categories. SNPs were categorized according their base exchange characteristics (transition vs. transversion); their genome position (noncoding vs. coding), their codon position (first, second, and third), and their coding influence (synonymous vs. nonsynonymous). In total, eleven, four, and ten SNPs in first, second, and third position were located in overlapping adjacent ORFs. (B) Prevalence of nonsynonymous SNPs in 103 ORFs according to their functional capacity, such as biological regulation, DNA replication, metabolic process, structural protein, virus transcription, and unknown function. Three hundred nine nonsynonymous SNPs in each ORF were calculated for respective SNP number per kbp. Vertical bar represents average amount of SNPs in each ORF per kbp with standard error (SEM). No significant difference was determined. (C) Prevalence of nonsynonymous SNPs in every ORF per kbp with SEM of a total of 60 with an annotated function and 43 with unknown function. The difference between two groups was compared using *t* test (*α *= 0.05). Different capital letters address the significant difference (*P *<* *0.05).

A global detection of CpGV polymorphisms was achieved by mapping reads of the twenty analyzed isolates to the common reference CpGV-M, thereby creating a link between the variable sites or SNP positions of each sequenced and analyzed CpGV isolate. The main focus was on the detection of SNP positions that could be used in further steps for the identification and quantification of CpGV isolates especially in non-homogenous or heterogenous CpGV populations. A total of 753 SNPs corresponding to 0.61 per cent of the entire CpGV-M genome sequence (123,529 bp) was detected in all aligned reads. The SNPs included 544 transitions and 209 transversions. Six hundred sixty-five SNPs (88.3%) were distributed over 124 ORFs, whereas 88 SNPs (11.7%) were found in noncoding regions (Fig. 1A). Only 18 ORFs were without any SNP and were thus fully conserved in all isolates (Supplementary Table S1). The annotations of these ORFs comprised functions in the oral infection process, viral replication as well as BV and ODV production (Supplementary Table S2) (Luque et al. 2001; Rohrmann 2019). Out of 665 SNPs in coding regions, 163 (eleven SNPs in overlapping ORFs) and 128 SNPs (four SNPs in overlapping ORFs) were located in first and second codon position, respectively, whereas 398 SNPs (ten SNPs in overlapping ORFs) were found in the third codon position; 355 and 309 SNPs were synonymous and nonsynonymous nucleotide changes, respectively (Fig. 1A). All CpGV ORFs were assigned to one of the following six functional groups: (1) biological regulation, (2) DNA replication, (3) metabolic process, (4) virus transcription, (5) structural proteins, and (6) ORFs with unknown function (Fig. 1B and Supplementary Table S2). The average SNP number per ORF with known function ranged from 2.75 (DNA replication) to 3.59 (biological regulation) per kbp, which was not significantly different from that in ORFs with unknown function with a slightly higher SNP density of 5.61 per kbp (*F*_5,97_ = 2.13, *P *=* *0.07) (Fig. 1B). A significant difference occurred in comparison of SNP density between all ORF with known and unknown function (*P *<* *0.05) (Fig. 1C), indicating high polymorphisms in ORFs of unknown function.

### 3.3 SNP mapping and genotype composition

**Figure 2.**
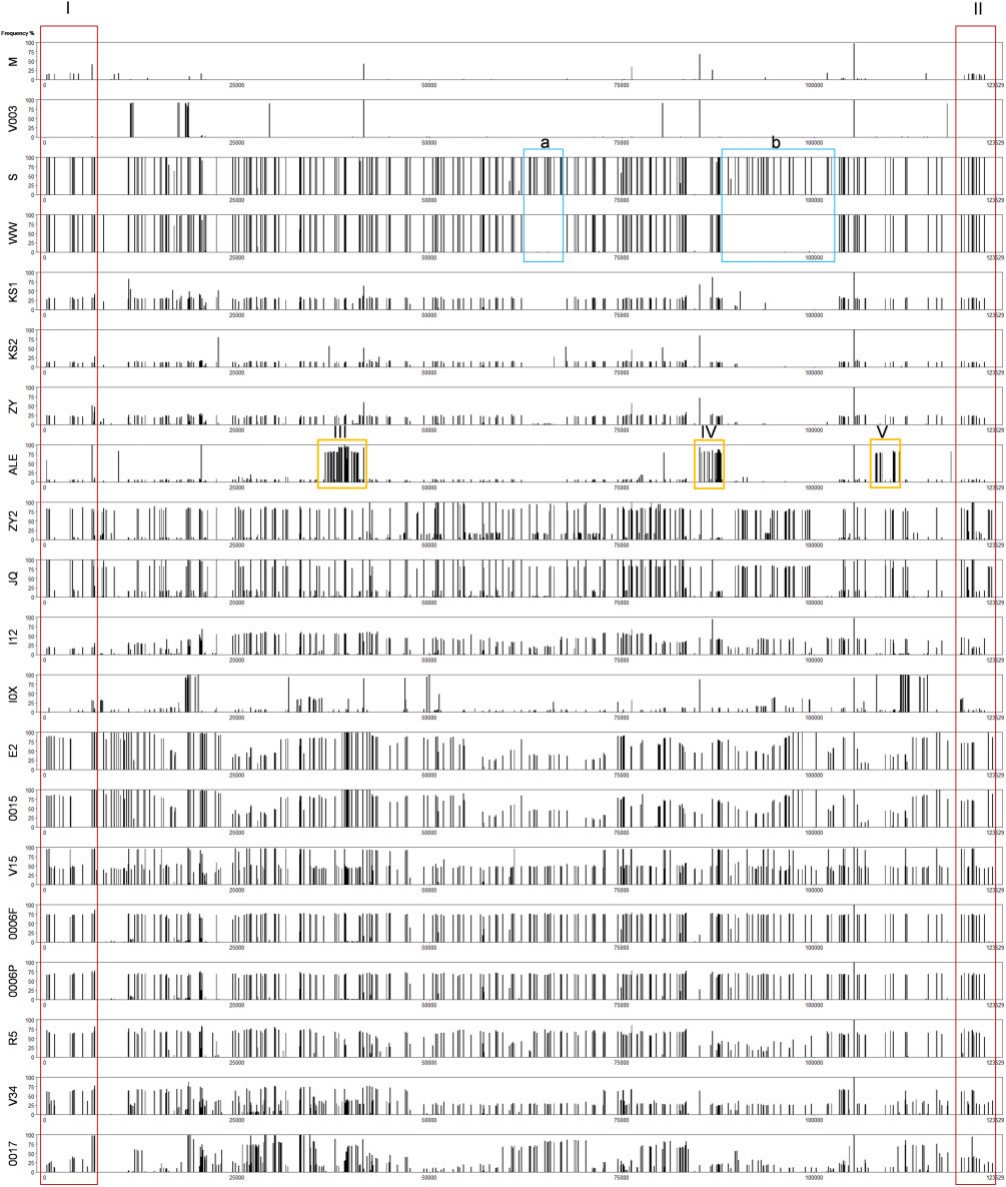
SNP distribution of CpGV isolates mapped against CpGV-M reference (GenBank KM217575). Re-sequencing of CpGV-M, -S, -E2. and -I12 revealed 51, 268, 282, and 393 SNPs, respectively, as well as 244 SNPs in CpGV-I0X. CpGV-ALE, -JQ, -KS1, -KS2, -ZY, -ZY2, and -WW contain 320, 475, 258, 250, 278, 445, and 246 SNPs, respectively. Isolates from commercial products, CpGV-V15, -R5, -0006P (Gueli Alletti et al. 2017) as well as CpGV-V003, -0006F, -0015, -V34, and -0017, contained 331, 281, and 377 as well as 51, 364, 273, 399, and 384 SNPs, respectively. SNP island I/II across all CpGV isolates is marked with red box. SNP hotspot regions III, IV, and V in CpGV-ALE and two stretches a and b in CpGV-WW are indicated by orange and blue boxes, respectively.

The SNP frequencies at all variable SNP positions of each CpGV isolate were plotted against the CpGV-M consensus sequence as reference (Fig. 2). The lowest number of variant SNP positions in field collected isolates was detected in CpGV-M (51 SNPs) (Fig. 2). For nine other field isolates the number of variant SNP positions varied from 200 to 400 per genome: CpGV-I0X (244), -WW (246), -KS2 (250), -KS1 (258), -S (268), -ZY (278), -E2 (282), -ALE (320), and -I12 (393) (Fig. 2). The highest number of variable SNP positions was found for CpGV-ZY2 (445) and CpGV-JQ (475) both belonging to genome group F (Fig. 2, Table 1). From the entity of the 753 variable SNP positions, 723 (96%) were isolate or group specific for the 12 field isolates, whereas the remaining 30 SNP positions were specific solely for the commercial CpGV isolates (Table 1). The 723 SNP positions were considered to reflect the natural polymorphisms in CpGV populations and therefore were used for the quantification and deciphering of CpGV population structures. For an in-depth population only a significant selection of isolate-specific and group-specific SNP positions were applied (Table 2) since most variable SNP positions were specific for all kinds of isolate combinations at low frequencies (Supplementary Table 3). Frequencies of specific SNP in genome group A (CpGV-M), genome group B (CpGV-E2), genome group E (CpGV-S), genome group F (CpGV-JQ and -ZY2), genome group G (CpGV-ALE), as well as combined genome group BDEFG (CpGV-WW, -ZY2, -KS2, -KS1, -ALE, -JQ, -ZY, -S, -I12, -E2) and DEFG (CpGV-WW, -ZY2, -KS2, -KS1, -ALE, -JQ, -ZY, -S, -I12), respectively, were applied to quantify the genotype composition of all isolates (Table 2, Supplementary Table S3). An in-detail analysis of the genotype composition of all CpGV isolates is given in the following sections.

Based on the quantitative SNP analysis applied in this study, most CpGV isolates were determined to be a mixture of two dominant genome groups, which was reflected by a typical pattern of three different SNP frequencies: SNP frequency for genotype a (ƒ_a_), frequency for genotype b (ƒ_b_), and isolate frequency ƒ_a_ + ƒ_b_ that was often ƒ_a+b_ = 1 when genotype a and b shared the same SNP position. Only CpGV-M, -S, -WW, and -V003 appeared to be highly homogenous showing few genomic variations (Fig. 2, Table 2).

#### 3.3.1 Homogenous population structure of reference isolate CpGV-M

The assembly of the re-sequenced isolate CpGV-M against its own reference revealed a low number of variable sites. Only 51 SNPs were identified, of which only five exceeded an alternative frequency of 20 per cent. In particular, only one SNP had a frequency above 95 per cent (at position 105,178 in ORF123). In the remaining 19 CpGV isolates, the alternative frequencies of this position ranged from 92 per cent to 100 per cent, strongly proposing that this position is most likely an annotation error in the consensus of the CpGV-M reference genome. On the other hand, the high accuracy of the reference sequence was confirmed by the absence of highly variable sites in this analysis (Fig. 2). It is important to mention that these observed 51 SNP positions were not specific for CpGV-M only, but most likely reflected the natural and internal variation of CpGV-M. For the detection of SNPs specific for only CpGV-M the sequencing results of all other isolates needed to be considered. In total, 58 SNP positions that were variable for all isolates except CpGV-M, were counted as CpGV-M specific and were later used for the quantification of CpGV-M (Fig. 3, Table 2) in the other analyzed isolates. These SNP positions were not visible in the alternative SNP frequency plot since these were identical to the reference CpGV-M itself. SNPs being specific for other isolates and genome groups were not detected, underscoring the homogeneity of CpGV-M (Table 2). Occurring SNP locations in CpGV-M were concentrated in ORF1 (*granulin*), ORF2, ORF6, ORF7 (*ie1*), ORF10 (*chitinase*), ORF139, ORF140 (*fgf-3*), and ORF141 (*egt*). An obvious SNP island I/II with a SNP frequency <5 per cent, covering 8.6 kbp from genome map position 119,522 to 6,149 was noted in the circular genome of CpGV-M (Fig. 2). These SNPs were also identified with variable frequency across all CpGV isolates except for CpGV-V003. Nonsynonymous SNPs were found in the genes of *granulin* (1 SNP), ORF2 (2), *pk-1* (1), ORF6 (2), *ie1* (1), ORF8 (1), *chitinase* (1), ORF139 (1), *fgf-3* (4) and *egt* (3).

**Figure 3.**
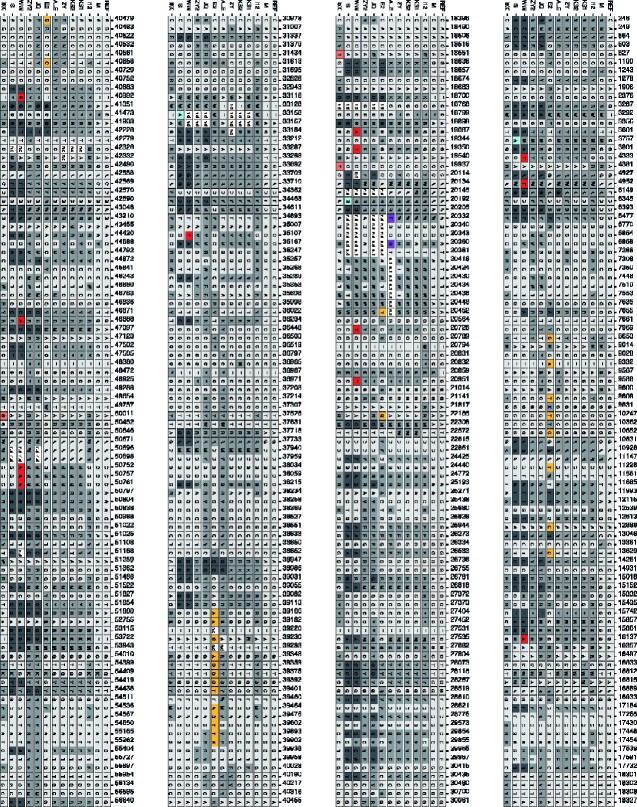
Detailed map of SNP positions of twelve CpGV field isolates that mapped against CpGV-M (GenBank KM217575) as reference (REF). Isolate specific variant sites (=unique for one isolate only), where all other isolates had either no base exchange or <100% in alternative variant at the same position were plotted in purple (CpGV-ALE), yellow (CpGV-E2), red (CpGV-WW), sky blue (CpGV-S), and dark orange (CpGV-I0X). Ambiguous SNPs displayed with IUPAC ambiguity codes are filled by dark gray. SNPs occurred in more than a single CpGV isolate as shown by unique A, T, G, or C are filled with light gray.

**Figure 3.**
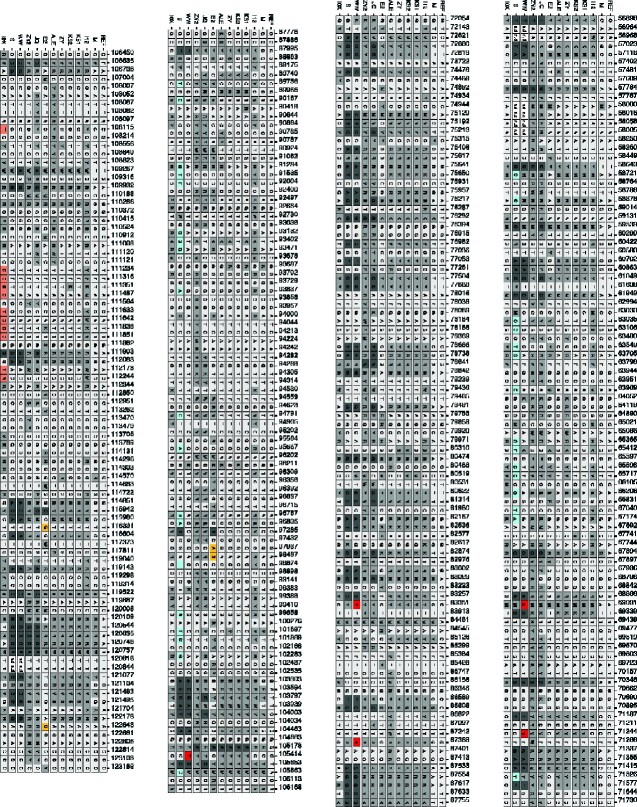


#### 3.3.2 CpGV-V003, a homogenous selection from CpGV-M

CpGV-V003, a resistance-breaking isolate selected from CpGV-M, also contained 51 SNPs. But only ten SNPs, at position 1,649, 11,147, 18,799, 20,332, 20,360, 2,859, 41,473, 76,292, 85,126 and 105,178, were identical to those positions found in CpGV-M (Fig. 2). The overall SNP pattern revealed 26 and 25 SNPs with an alternative SNP frequency of <6 and >73 per cent, respectively. With its unique SNP pattern it appeared to be very homogenous. The 58 CpGV-M specific SNP positions identified V003 as highly similar to CpGV-M (Table 2) with a slightly different SNP pattern. SNPs were concentrated in mainly ORF15 and ORF22-24, whereas the SNP island I/II was missing.

#### 3.3.3 CpGV-WW and -S (genome group E) with identical SNP pattern

The overall SNP patterns of CpGV-WW and -S were highly identical in their location and frequency (Fig. 2). The majority of alternative SNP frequencies of CpGV-WW and -S in relation to CpGV-M were more or less equally close to 100 per cent identifying them as homogenously different to CpGV-M. Only 18 of 246 SNP positions in CpGV-WW and 18 of 268 SNP positions in CpGV-S were far <100 per cent. Two major regions with a significant difference between CpGV-S and -WW were found in genome region at positions from 63,030 to 67,744 and from 88,863 to 102,535; here CpGV-WW lacked specific SNPs present in -S, and the -WW sequence was identical to that of the CpGV-M reference without any of the SNP variants. These sequence stretches were termed CpGV-WW fingerprint regions (a) and (b) (Fig. 2). From all 246 and 268 SNP positions of CpGV-WW and -S, respectively, no SNP position was detected to be only specific for one or both isolates. The half of CpGV-WW and -S SNPs was further specific for isolate groups CpGV-KS1, -KS2, -ZY, -ZY2, -JQ, -ALE, and -I12 (45 SNP positions, Table 2) as well as for isolate groups -KS1, -KS2, -ZY, -ZY2, -JQ, -ALE, -I12, and -E2 (75 SNP positions, Table 2). These two groups of SNPs were characterized as suitable for CpGV-WW and -S quantification for three reasons: (1) the SNP frequency was 100 per cent for these two groups in CpGV-WW and -S only; (2) the genome-wide characteristic SNP pattern of CpGV-WW (including the -WW fingerprint regions a and b) was found in -KS1, -KS2 and -ZY, but at lower frequencies, identifying these isolates as mixtures with -WW (see analysis below); (3) the difference between combined genome group of BDEFG and DEFG is the presence of the shared CpGV-E2 (group B), which allowed for quantification of the composition of combined genome group BE and specific genome group E, respectively.

#### 3.3.4 Mixed isolates of genome groups A and E found in CpGV-KS1, -KS2, and -ZY

The three isolates CpGV-KS1, -KS2, and -ZY exhibited a similar SNP pattern in their position and frequency (Figs. 2 and 3). No SNP position specific for solely one of these three isolates was found (Fig. 3). The similarity of these three isolates was further reflected by the total number of SNPs found: 258, 250, and 278 for CpGV-KS1, -KS2, and -ZY, respectively (Fig. 2). When mapped against the CpGV-M reference, the genome-wide SNP density and abundance resembled the picture of CpGV-WW (including fingerprint region a and b) indicating a common composition. Based on the 58 CpGV-M specific SNP positions the median proportions of 69, 86, and 77 per cent of CpGV-M within these field isolates were calculated (Table 2). In addition to CpGV-M SNPs, there were only the CpGV-WW and -S specific SNPs in two combined genome groups BDEFG and DEFG, which could be used for quantification of CpGV-WW at a proportion of about 29 to 30 per cent for CpGV-KS1, 14 per cent for -KS2 and 22 per cent to 23 per cent for -ZY (Table 2). Due to the indicated presence of the CpGV-WW fingerprint regions a and b, the mixtures of these three field isolates were characterized as mainly CpGV-M and a minor part of CpGV-WW.

#### 3.3.5 CpGV-I12 and -I0X with geographic SNP fingerprints

The two Iranian isolates CpGV-I12 and CpGV-I0X differed in their SNP abundance and density. CpGV-I12 was identified as a mixture of CpGV-M (56%) and CpGV-S (42–46%) according to CpGV-M and -S specific group SNPs, though frequencies of CpGV-M specific SNP showed a highly uneven distribution. The CpGV-WW fingerprint regions a and b were lacking. CpGV-I0X was measured to only consist of mainly 93 per cent CpGV-M (Table 2). Except for the quantification based on CpGV-M and -S, an additional 24 SNPs specific for -I12 and -I0X were detected with frequencies ranging from 2 per cent to 35 per cent for CpGV-I12 and -I0X (Fig. 2, Table 2). Especially for CpGV-I0X, these specific SNP frequencies ranged from 4 per cent to 100 per cent representing an Iranian geographic fingerprint and the main differences to CpGV-M (Table 2).

#### 3.3.6 CpGV-ALE is similar to CpGV-M with distinguishing features

A total number of 320 variant SNP positions were detected for CpGV-ALE, the reference isolate of genome group G (Fan et al. 2020b). Ninety-six SNPs had a frequency above 50 per cent, represented by three SNP clusters around genome position 36,000, 86,000, and 108,000, and termed SNP clusters III, IV, and V in Fig. 2. Twenty-one SNP positions were detected to be solely specific for CpGV-ALE and 22 positions being group-specific for CpGV-ALE and -JQ (Table 2). Both, the 21 and 22 specific SNPs were located within the three SNP clusters III, IV, and V with SNP frequencies above 50 per cent, representing fingerprint SNPs of CpGV-ALE (Fig. 2). Based on the CpGV-M specific SNP frequencies, the CpGV-ALE was represented by CpGV-M by about 93 per cent and a smaller amount of about 6 per cent CpGV-S (Table 2). According to the analysis, CpGV-ALE appeared to be similar to CpGV-M but with a characteristic unique fingerprint represented by the specific clusters III to V, which were solely present in CpGV-ALE specific SNPs.

#### 3.3.7 CpGV-JQ and -ZY2 with fractions of CpGV-WW and CpGV-M

CpGV-JQ and -ZY2 shared 239 SNPs with -WW, echoing that genome group E was within these isolates. A total number of 89 SNP positions were identified to be specific for CpGV-JQ and CpGV-ZY2 only, representing their own genetic marker for genome group F (Fan et al. 2020b). According to these group F specific SNPs, these isolates were not homogenous but a mixture, which were also visible by two major SNP frequency groups (Fig. 2). Groups B, D, and G could be excluded to be part of CpGV-JQ and CpGV-ZY2 since frequencies for their isolate-specific SNPs were not measured (Table 2). In conclusion, the SNPs of the two combined genome groups BDEFG and DEFG could be reduced to the specificity for E and F (EF) (Table 2). For CpGV-JQ, the SNP frequency in group F was similar with that in group BDEFG (75 specific SNPs) but different from DEFG (45 specific SNPs), indicating that 79 per cent genome group F was represented by genome group BDEFG, whereas 17 per cent genome group E was indicated by genome group DEFG. 15 per cent CpGV-M (group A) and 77 per cent genome group F was also identified in -ZY2 (Table 2). It was difficult to determine the exact genomic proportion of CpGV-WW, but ranging from 5 to 42 per cent.

#### 3.3.8 CpGV-E2 and -0015 with highly heterogenous SNP patterns

CpGV-E2 and -0015 were unique as they showed a highly uneven SNP frequency distribution, resembling a ‘wave’-like pattern, clearly visible for the alternative SNP frequencies between genome positions from 10,000 to 113,000. Another characteristic was the presence of two cluster regions with SNP frequencies of 100 per cent at genome positions (1) 10,000 to 13,000 and (2) 39,000 to 43,000. Despite the heterogenous SNP pattern, a total number of 68 for only CpGV-E2 specific SNP positions was detected (Table 2). These specific SNPs were used to calculate the median presence of CpGV-E2. Its quantification is hampered by its genotype heterogeneity, which is reflected by the self-quantification with median proportion of 84 per cent (28–100%) by the CpGV-E2 specific SNPs only. When the 75 SNPs specific for isolate groups CpGV-WW/-S and -E2 were used for quantification, a median proportion of 67 per cent (3–100%) was measured. However, the 45 group specific SNPs for CpGV-WW/-S without -E2 was zero and therefore could not prove the presence of any proportion of CpGV-WW or -S in CpGV-E2 (Table 2). Except for the 58 CpGV-M specific SNPs with a frequency of up to 20 per cent (0–98%) no other specific SNP frequencies could be calculated (Table 2), hinting that it was a mixture of CpGV-M, but difficult to quantify due to the range of the CpGV-M specific SNP frequencies.

#### 3.3.9 CpGV commercial isolates

The isolate CpGV-0015 was a commercial isolate derived from *in vivo* propagation of CpGV-E2. SNP positions and frequency of both isolates of CpGV-E2 and -0015 were identical (Figs 2 and 3). CpGV-R5, -0006F, and -0006P showed the similar SNP pattern as CpGV-I12, of which all were mixtures of CpGV-M and -S. Nine and fourteen SNPs specific for CpGV-S were identified in CpGV-WW fingerprint regions a and b, respectively, indicating that these isolates contain genome group E virus similar to CpGV-S but not to CpGV-WW. SNP frequency of identical SNP positions in CpGV-0006P and -0006F were used to evaluate the genotype proportion from different batches of production (Figs. 2 and 3).

CpGV-V15 comprised 42 per cent CpGV-E2 based on the CpGV-E2 specific SNPs (Table 2) and 49 per cent CpGV-S based on the CpGV-WW/-S group-specific SNPs that did not include CpGV-E2 (Table 2). Consequently, the 75 CpGV-WW/-S group-specific SNPs that were further specific for CpGV-E2 showed a proportion of 93 per cent indicating a mixture of CpGV-E2 and CpGV-S (Table 2) in CpGV-V15. Besides the 58 SNP positions specific for CpGV-M with a frequency of 7 per cent (3-53%), no other isolate was detected (Table 2). The difficulty in the quantification of CpGV-E2 due to its high heterogeneity was reflected by 5 to 95 percentiles ranging from 0 to 48 per cent in CpGV-V15 as well as CpGV-E2 with 28 to 100 per cent (Table 2).

CpGV-V34 and -0017 also appeared to have an uneven frequency distribution of SNPs. Since no specific SNPs from genome groups D, F, and G were identified in these isolates, the specific genome group combination of BDEFG and DEFG were thus collapsed as groups BE and E respectively. Both isolates contained SNP signals of genome group E were then 28 per cent and 19 per cent in V34 and 0017, respectively. Genome group A was presented in CpGV-V34 and -0017 with 38 per cent and 61 per cent as well. In consideration of the above group E proportion, the group B proportion of CpGV-V34 and -0017 was thus 39 per cent and 11 per cent respectively, derived from the specific genome group combination BE (Table 2).

### 3.4 Indels/*pe38* repeat motif

**Figure 4.**
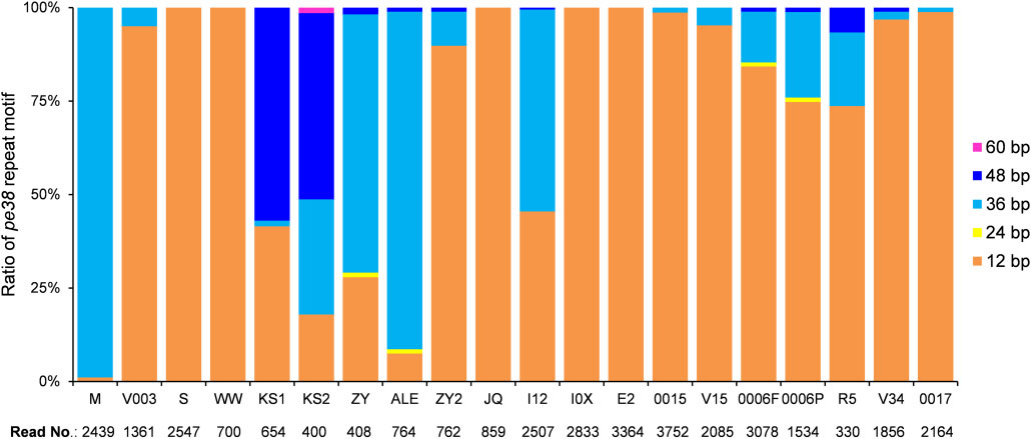
Percentage of five types of 12 bp repeat variants in *pe38* in twenty CpGV isolates. Percentage and amount of reads were obtained from read counting methods.

Due to its significance in overcoming CpGV resistance, the number of the 12 bp repeat motif GACACAGTGGAT within *pe38* was determined from the quality-passed reads to evaluate its genetic variation for each isolate. Based on the reads that comprised the entire motif and identified by unique adjacent marker sequences, between 400 (CpGV-KS2) and 3,752 (CpGV-0015) reads were used for this analysis. Between one to five copies of the 12 bp repeat motifs (1–5 ***×*** 12 bp) were identified in the *pe38* of the twenty CpGV isolates (Fig. 4), which was consistent to the previously detected repeat motifs (Fan et al. 2020b). Interestingly, the 1 ***×*** 12 bp repeat motif was found in all twenty isolates at variable proportions. Even the re-sequenced CpGV-M harbored a minor portion (0.1%, 3 reads) of the 1 ***×*** 12 bp repeat motif. Its abundance increased to 95 per cent (1,293 reads) in CpGV-V003, a selection from CpGV-M, whereas the rest of the genome sequences of CpGV-M and -V003 showed highly similar SNP patterns (Fig. 2). The 3 ***×*** 12 bp repeat was also present to a significant level in CpGV-KS2, -ZY, -ALE, -ZY2, -I12, -0006F, -0006 , and -R5. The presence of a 4 ***×*** 12 bp repeat motif was typical for CpGV-KS1 and -KS2 as well as to a lower extent to several other isolates, whereas a 5 ***×*** 12 bp repeat motif was only found in CpGV-KS2.

### 3.5 HC on principal components

**Figure 5.**
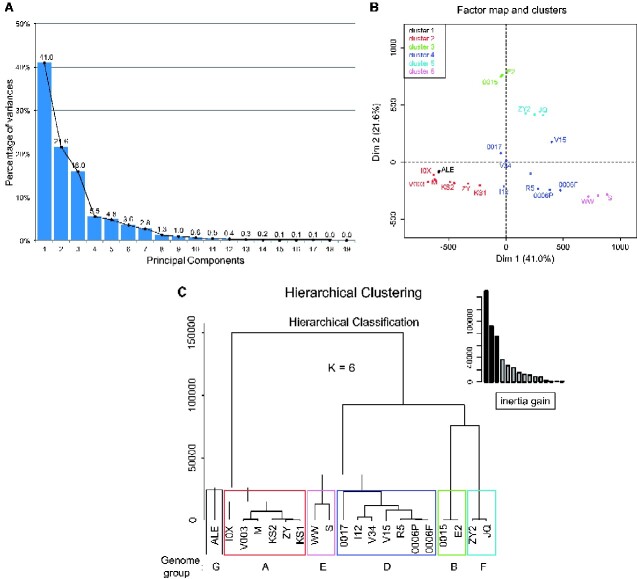
HCPC analysis of twenty CpGV isolates based on identified SNPs data set. (A) Percentage of variance of each component; (B) relative position of twenty CpGV isolates in a two-dimensional factor map evaluated by PCA. Different colored dots and open rectangles indicate individual isolate and isolate cluster center, respectively; (C) the first seven components was used for HC instead of nineteen suggested by PCA and six clusters were generated based on the previously proposed genome groups A, B, D, E, F, and G rather than four clusters recommended by inertia gain.

Consensus sequence-based phylogenies can properly reflect the relationship of isolates only if the isolates are homogenous. In case of high sequence heterogeneity and/or mixtures of different genotypes as observed for most of the studied isolates, a consensus sequence ignores existing variation within a baculovirus population. To overcome this limitation, here an HCPC based on the SNP frequency and position was applied to estimate the similarity among the different CpGV isolates. PCA and HC are complementary methods to cluster individual samples. Since the first seven principal components (PC) covered 95.2 per cent information (variances) presenting in the SNP data set and excluded the noise from data (Fig. 5A), an individual (isolate) factor map was drawn on a plot that corresponded to the spatial relative position of each isolate (Fig. 5B). The hierarchical tree suggested four clusters because the inertia gain to more clusters was minor. For further analyses, however, it was decided to use six clusters on the basis of previously identified genome groups A–G. The highly homogenous isolates CpGV-M and V003 were found in the bottom left of the quadrant and CpGV-I0X, -KS1, -KS2, -ZY, and -ALE containing the major genotype from genome group A (CpGV-M) were closer to them (Fig. 5B and C). In contrast, the highly homogenous isolates CpGV-WW and -S were located at the right bottom of the quadrant and CpGV-0006P, -0006F, -R5 containing the major genome group E (CpGV-S) were closer to them. Highly heterogenous CpGV-E2, -I12, -0015, and -0017 were located nearly on the axis of the first principal component except for CpGV-V34 which was composed of almost one third of genome group A (38%), genome group B (28%), and genome group E (39%) and was located in the middle of these groups; CpGV-V15 was also located in the middle between genome group E and genome group B as it was composed of nearly 50 per cent CpGV-E2 and 50 per cent CpGV-S (Fig. 5B and C). The genetic compositions of twenty analyzed CpGV isolates represented by their SNPs were reflected by their relative position in the HCPC plot, where the mixture isolates of CpGV-were located between homologenous CpGV-M and CpGV-WW/-S (Fig. 5B).

## 4. Discussion

High throughput sequencing methods with high genome coverage and read depth provide new tools to study the population structure of virus isolates. As a consequence, more comprehensive genetic information can be unveiled than in previous studies. The data sets of re-sequenced genomes of CpGV-M, -S, -E2, and -I12, with mean read depth from 3,320 to 4,045 was between 100 and 1,000-fold higher than previous data sets of these viruses, ranging from 3.9- to 243-fold coverage (Wennmann et al. 2017). This high sequencing coverage allows determining the location of SNPs as well as their frequency distribution. Previous SNP analyses of CpGV-M, -S, -E2, -I12, -I07, representing the genome groups A to E, rendered between 2 and 356 group or isolate specific SNPs (Wennmann et al. 2017). The inclusion of seven new Chinese isolates and application of the consensus-free method extended the pictures of CpGV diversity to seven groups (A–G) (Fan et al. 2020b). It also revealed, however, that a number of SNPs, previously considered as genome group specific, were either present in isolates classified into different groups or not present in closely related isolates belonging to the same phylogenetic lineage, suggesting that these SNPs were rather isolate specific (Wennmann et al. 2017; Fan et al. 2020b). By using NGS data sets of twenty CpGV genomes, different phylogenetic lineages of CpGV could be re-evaluated on the basis of the distribution of isolate and group specific SNPs (Table 2). In consequence, the re-examination and identification of group-specific SNPs is thus much more robust than in previous studies with five genomes, when each phylogenetic genome group was based on a single consensus sequence. In addition, SNP detection obtained directly from aligned reads from NGS data rather than consensus sequence alignments can generate a much more complete picture of nucleotide variations, since minor SNP frequencies are noted and not neglected. Therefore, it is suggested that SNP screening in baculovirus sequence analysis needs to be done before consensus sequence is generated. SNP density showed a significant difference between pooled ORFs with known function and pooled ORFs with unknown function, revealing considerable variability in ORFs of unknown function. Because most CpGV ORFs are annotated on the basis of gene function studies of AcMNPV as well as other GVs and NPVs, they are relatively conserved in baculoviruses in contrast to ORFs of unknown function that are newly integrated into the CpGV genome and showing more recent.

Another resource of mutations in CpGV genomes are the indels. The number of the 12 bp repeat motif in *pe38*, corresponding to the ability to overcome type I resistance in CpRR1, was quantified. When correlating bioassay data (Fan et al. 2020a) with the distribution of 1 **×** 12 bp repeats in different CpGV isolates (Fan et al. 2020b), a relation between a resistance-breaking activity (fraction of 1 **×** 12 bp repeats larger than 46%) and a non-resistance-breaking activity was underlined (Graillot et al. 2016; Gueli Alletti et al. 2017; Jehle et al. 2017; Sauer et al. 2017a; Fan et al. 2020b). CpGV-M and -ALE were the least active isolates in CpRR1 (Fan et al. 2020a) and were the only isolates with a very low percentage of 1 **×** 12 bp repeats supporting the *pe38* model of resistance-breaking isolates (Gebhardt et al. 2014). This model is now further supported by sequencing of CpGV-V003 that can overcome type I resistance and was a laboratory selection from CpGV-M (Zingg 2008; Zingg et al. 2011). As demonstrated by the sequencing data and SNP analysis, CpGV-V003 was similar to CpGV-M, except that a 95 per cent portion of the reads covering the repeat region of *pe38* contained only a 1 **×** 12 bp repeat motif as is typical for CpGV isolates overcoming type I resistance. Since CpGV-M contained a minor population with the 1 **×** 12 bp repeat, this portion was apparently selected and enriched to obtain CpGV-V003.

On the other hand, only ten SNP positions were shared between CpGV-M and CpGV-V003, also excluding those from SNP island I/II, proposing that they were lost during the selection process, while nucleotide frequency at other SNP positions were enriched or new SNPs were selected, for example in the CpGV-V003 specific SNP hotspot covering *orf17R* (ORF22), *pep* (ORF23), and *pe38* (ORF24). As most nucleotide frequencies of CpGV-V003 were close to 100 per cent, it can be concluded that the virus selection successfully resulted in a notably pure genotype that was even more homogeneous than the original CpGV-M.

The SNP pattern of CpGV-WW was highly similar to CpGV-S. The most obvious differences between both viruses were the CpGV-WW fingerprint regions a and b, covering a total number of 30 SNPs present in CpGV-S but not in -WW. As these regions are apparently shared between CpGV-WW and -M, it is plausible to assume that they are the result of a recent recombination event between CpGV-WW and CpGV-M or its ancestors. The CpGV-WW fingerprint regions a and b are located in *p45*, *dnapol*, *desmoplakin*, *lef3*, *iap5*, *lef9*, *dna-ligase*, which are supposed to be involved in viral DNA replication and BV production (Supplementary Table S2), in which the SNPs result in amino acid sequence changes and may cause virulence differences between the two viruses observed in infection experiments of larvae with type I (CpRR1) and type II (CpR5M) resistances are of interest (Fan et al. 2020a). When the genotype attribution of the seven Chinese CpGV isolates (Fan et al. 2020b) is considered, a similar genetic composition of CpGV was determined, irrespective of whether the genome group specific SNP positions from consensus sequences or from new consensus-independent SNP positions were applied for quantification. Thus, the previous approach also provided reliable results, although at a lower level of accuracy since the consensus sequence lost minor variants in genome sequence prior to SNP determination.

Both CpGV-E2 and -0015, of which the latter is a commercial production batch of CpGV-E2, were different propagations of the same virus had almost identical SNP distribution and frequencies (Fig. 2). The frequencies of their SNPs could not be grouped into one, two, or three majority classes as it was possible for other pure genotype isolates or mixtures of them. Their SNP frequencies occurred in a highly uneven distribution suggesting that CpGV-E2 consists of unusually manifold genotypes, adding to the “wave”-like distribution patterns of SNPs (Fig. 2). Similar ‘wave’-patterns, though to a lower degree and at different SNP positions, were also noted for CpGV-I12, -R5, and -0017. There is no obvious reason which could explain such patterns. But it is striking that CpGV-E2 and -0015 showed virtually the identical SNP frequency pattern, although they were independently propagated in different laboratories, starting from the same virus inoculums. This finding can only be explained by the existence of selection constraints in CM larvae and/or in CpGV-E2 resulting in the stabilization of the complex composition of this virus.

Interestingly, CpGV-E2 was shown to be one of the most virulent CpGV isolates, being infective for all types of CpGV resistance I to III (Gueli Alletti et al. 2017; Sauer et al. 2017b). It can be speculated that CpGV isolates with such an internal heterogeneity are the most potent ones, suggesting that stable and heritable ‘heterosis’ is essential for their high virulence. It was previously noticed that genotypes of CpGV-E2 could not be plaque purified (Winstanley and Crook 1993). This observation might be explained by the highly complex composition and potentially co-acting genotypes of CpGV-E2. Similarly, CpGV-0006P and -0006F were from different production batches using the same virus inoculums, which were essentially mixtures of two viruses (similar to CpGV-M and -S).

As the quantitative distribution of SNPs types was highly similar in both preparation (Fig. 2, Table 2), it can be concluded that the population structure of these genotype mixtures is also highly stable during *in vivo* propagation. Only new genetic host background, for example, resistant host individuals, may change this stable composition (Berling et al. 2009; Graillot et al. 2014, 2016, 2017, 2019). Our first findings that even complex compositions of virus mixtures can be stably propagated are an important issue for quality control of commercial baculovirus production, since it demonstrates the identity of such product compositions can be stably produced. NGS techniques as developed here and applied on production batches will allow easy and straight forward tools for quality control of serial passages of commercial isolates consisting of mixed genotypes. As shown in Fig. 5, our results indicated that nine out of twelve naturally occurring CpGV isolates contain mixed and heterogenous genotypes. It is reported that other wild CpGV isolates were composed of variable genotypes in restriction endonuclease analysis (Rezapanah et al. 2008). Similarly, also other baculoviruses were found to contain variable genotypes in the field isolates (Erlandson 2009). Therefore the hypotheses can be laid down that field baculoviruses are evolving in ‘panmixia’, which allow different genotypes to recombine and interact with each other, contributing to the genetic diversity in baculovirus populations. The CpGV-WW fingerprint regions a and b are proposed to be the result of recombination with a genotype similar to CpGV-M.

Wild-type CpGV isolates originating from natural CM populations are in their majority genotype mixtures. Field-obtained CpGV-E and NPP-R1 were comprised of at least two genotypes, one of which is most likely identical to CpGV-M (Crook et al. 1985; Berling et al. 2009). Similar is found in other baculoviruses. Inheritable compositions of virus mixtures were highly prevalent in Spodoptera frugiperda multiple nucleopolyhedrovirus (SfMNPV), ensuring highly genetic diversity and infective activity in its field geographic populations (Escribano et al. 1999; Simón et al. 2004; Barrera et al. 2011). On the other hand, *Pieris brassicae* larvae infected either with Artogeia rapae granulovirus 1 (ArGV1) or with other ArGV strains, revealed in most cases the presence of ArGV1 or recombinants between inoculums and ArGV1, suggesting ArGV1 is present as latent genotype in the host population (Smith and Crook 1993). For wild-type Lymantria dispar multiple nucleopolyhedrovirus (LdMNPV) it was shown by physical mapping that they were comprised of three and eight distinct genotypes, respectively (Smith and Crook 1988; Harrison et al. 2016). Either the same or different inoculum doses of Helicoverpa armigera nucleopolyhedrovirus (HearNPV) could induce the genetic diversity in HearNPV populations (Baillie and Bouwer 2013; Kitchin and Bouwer 2018). Besides that, host and environmental stress were correlated with genetic diversity of HearNPV, which is presumably caused by activation of latent viruses (Moscardi 1999). A similar observation, validated by NGS analyses, was recently noted for PhopGV, when infection of *Phthorimaea operculella* larvae with the isolate PhopGV-GR3 resulted in the activation of a latent virus PhopGV-R (Larem et al. 2019b). As proposed for SfMNPV, genotype mixtures of wild-type viruses might trigger a mutualistic interaction between genotypes, resulting in an increased virulence compared to a single genotype (Simon et al. 2006; Lopez-Ferber et al. 2003). The co-occlusion of different genotypes in the occlusion bodies of nucleopolyhedroviruses may reflect a morphological adaptation to facilitate preservation of such heterogeneity in nature (Moscardi 1999; Cory and Franklin 2012). The observed high mortality in codling moth resistance test induced by a mixed and heterogenous genotype of CpGV isolates containing resistance-breaking markers, derived from either wild-type isolations or commercial selected approaches, were in line with the aforementioned findings that mixed genotypes of baculoviruses are more virulent against their host (Graillot et al. 2016; Gueli Alletti et al. 2017; Jehle et al. 2017; Sauer et al. 2017a; Fan et al. 2020b).

The PCA, the first step of HCPC, is a mathematical procedure to reduce a multiple dimension problem to a lower number of dimensions while preserving the main information of the original data. In this study, it was applied as a pre-processing step. The first two and three components explained 60 per cent and 80 per cent of the data distribution, respectively (Fig. 5A). PCA is a standard procedure in analyzing RNA sequencing data where it is used to visualize and explain the reliability of biological replicates and the effect of treatments (Treutlein et al. 2016; Xing et al. 2017; Xue et al. 2012). HC based on the multidimensional variance (i.e. inertia) is used to generate hierarchical trees. These two methods complement each other as it is in the HCPC method, which can be applied on the frequency of SNP positions without any previous knowledge about the isolates’ genetic composition and allows a reliable grouping. This is in contrast to the common analysis based on consensus sequences that are drawn from majorities of every single base in the read assembly reducing the information to the most frequent occurring nucleotide or nucleotide ambiguities. As a result of applying HCPC, an imaginary triangle can be drawn between genome groups A (CpGV-M) and genome group E (CpGV-WW and -S), and group B (CpGV-E2, 0015) (Fig. 5B and C). Within this triangle, the isolates were arranged according to their corresponding mixtures of genotypes related to group A, E, and B. Genome clustering based on the SNP variants is not equivalent to phylogenetic analysis. Phylogenetic tools are often neither appropriate nor practical to analyze genetic heterogeneity of a given isolate consisting of a mixture of two or more homogenous genotypes or of highly heterogenous genotypes. Alternatively, HCPC is a procedure capable of clustering baculovirus isolates into different genotype clusters to assess the (dis)similarity as well as relationship from one isolate to another. Here, the quantitative composition, distribution, and frequency of SNPs are used to evaluate the genetic distance among different isolates in its full complexity. Thus, HCPC analyses may become an important and expedient tool to characterize the population structure of virus isolates for different purposes, such as isolate characterization, population dynamics but also for quality control and registration purposes for baculoviruses used as biological pest control agents.

## Supplementary data

Supplementary data are available at *Virus Evolution* online.

## Data availiability

The raw SNP counts and frequencies that were used in this study have been provided in the Supplementary Data (Supplementary Table S4).

**Conflict of interest:** None declared.

## Supplementary Material

veaa073_Supplementary_Data
