# Supplementary material for: Population structure of Cydia pomonella granulovirus isolates revealed by quantitative analysis of genetic variation
Source: Virus Evol. 2020 Sep 29;7(1):veaa073. doi: 10.1093/ve/veaa073 (PMC7816688; doi:10.1093/ve/veaa073)
Supplement: veaa073_Supplementary_Data [file veaa073_supplementary_data.zip › Tables S1-S3.docx]

**Supplementary Table S1.** Eighteen conserved ORFs in the CpGV genome without SNP variants.

| ORF number in CpGV | Gene name | Transcription categories^#^ | Function description^*^ | Reference to function |
| --- | --- | --- | --- | --- |
| 5 | *orf5* | n.a. | n.a. | n.a. |
| 9 | *ac145* | early, late | Plays a role in oral infection; chitin binding activity; may interact with chitin synthesizing cells | (Lapointe et al., 2004; Wang and Zhang, 2006) |
| 40 | *orf40* | early | n.a. | n.a. |
| 52b | *ac106/107* | late | Essential gene for virus spread in cells | (Ono et al., 2012) |
| 54 | *ubiquitin* | early, late | Associated with BV production; involved in regulating the fate of nucleocapsids to form BV or ODV | (Biswas et al., 2018; Guarino, 1990; Reilly and Guarino, 1996) |
| 59 | *sod* | late | Converting superoxide induced by baculovirus infection into hydrogen peroxide, facilitating virus infection | (Bergin et al., 2005; Tomalski et al., 1991) |
| 65 | *Ac79* | late | Associated with ODV; involved in BV production and efficiency | (Braunagel et al., 2003; Ono et al., 2012; Wu and Passarelli, 2012) |
| 67 | *orf67* | late | n.a. | n.a. |
| 78 | *orf78* | early | n.a. | n.a. |
| 82b | *orf82b* | early | n.a. | n.a. |
| 86 | *p6.9^$^* | late | DNA binding protein | (Lai et al., 2018; Wilson et al., 1987) |
| 98 | *ptp-2* | early | pro-apoptotic effects of PTP2 involved in viral inclusion body yield | (Han et al., 2018) |
| 107 | *Ac76* | early, late | associated with the envelope of BV and ODV | (Guo et al., 2017; Wei et al., 2014) |
| 118 | *fp25k* | late | Structural protein of BVs and ODVs; deletion results in few polyhedra phenotype | (Garavaglia et al., 2012; Rosas-Acosta et al., 2001) |
| 133 | *orf133* | early | n.a. | n.a. |
| 136 | *orf136* | late | n.a. | n.a. |
| 137 | *lef-10* | early, late | Required for viral replication; behaving as prion | (Nan et al., 2019; Xu et al., 2016) |
| 142 | *orf142* | early | n.a. | n.a. |

# transcription categories are classified by promotor in individual ORFs (Pietruska, 2018). * gene function is described on the basis of homologous genes in other well-studies baculoviruses. $ baculovirus core gene. n.a., not available.

| **Supplementary Table S2.** Annotation of 103 ORFs-harbored SNPs. Based on the annotation reviewed by (Luque et al., 2001; Rohrmann, 2019). Identified SNPs were classified into five groups of biological regulation, DNA replication, metabolic process, structural protein, virus transcription. | | | | |
| --- | --- | --- | --- | --- |
| ORF | gene | function | Annotation | |
| 24 | *pe38* | delay DNA replication and synthesis | biological regulation | |
| 94 | *iap* | inhibitor of apoptosis | biological regulation | |
| 140 | *fgf-3* | vFGF initiate a cascade of events to accelerate systemic infection | biological regulation | |
| 17 | *iap-3* | inhibitor of apoptosis | biological regulation | |
| 102 | *tlp20* | BV production and DNA replication delay | biological regulation | |
| 76 | *fgf-1* | vFGF initiate a cascade of events to accelerate systemic infection | biological regulation | |
| 13 | *gp37* | enhance per os infection | biological regulation | |
| 123 | *fgf* | accelerate the establishment of systemic infections | biological regulation | |
| 143 | *me53* | BV and ODV associated | biological regulation | |
| 41 | *lef-2* | DNA primase accessory factor | DNA replication | |
| 58 | *lef-11* | DNA replication | DNA replication | |
| 113 | *lef-3* | single-stranded DNA binding protein | DNA replication | |
| 81 | *dbp* | DNAbinding protein, production of nucleocapsids and virogenic stroma | DNA replication | |
| 125 | *alk-exo* | an exonuclease activity, involved in DNA recombination | DNA replication | |
| 111 | *dnapol* | DNA polymerase | DNA replication | |
| 90 | *helicase* | DNA replication | DNA replication | |
| 74 | *lef-1* | DNA primase | DNA replication | |
| 128 | *rr2a* | nucleotide metabolism_ribonucleotide reductase subunits | metabolic process | |
| 10 | *chitinase* | degrade chitin | metabolic process | |
| 117 | *rr1* | nucleotide metabolism_ribonucleotide reductase subunits | metabolic process | |
| 141 | *egt* | inactivating insect molting hormones | metabolic process | |
| 11 | *cathepsin* | a metalloprotease along with chitinase | metabolic process | |
| 66 | *ptp-2* | pro-apoptotic protein | structural Protein | |
| 35 | *pif-3* | per os infection factor | structural protein | |
| 46 | *mp-nase* | assisting in viral transmission | structural protein | |
| 22 | orf17R | baculovirus PEP N domain | structural protein | |
| 73 | *38.7K* | nonessential structural protein and encodes a late gene | structural protein | |
| 23 | *pe/pp34* | polyhedron envelope protein | structural protein | |
| 47 | *p13* | Killing-associated gene | structural protein | |
| 56 | ac108 | associated with the PIF complex | structural protein | |
| 48 | *pif-1* | oral infection factor | structural protein | |
| 89 | *pif-4* | per os infectivity factor | structural protein | |
| 14 | *odv-e18* | ODV envelope fraction | structural protein | |
| 101 | *vp91* | structural protein | structural protein | |
| 96 | *vp39* | structural protein | structural protein | |
| 112 | *desmoplakin* | associated with BV and ODV (egress of virions from nuclei) | structural protein | |
| 83 | *p45(p48)* | essential for generation of perfect ODV and BV | structural protein | |
| 31 | *f-protein* | glycosylated and associated with the envelope of BV | structural protein | |
| 93 | *p33* | associated with BV and ODV | structural protein | |
| 37 | *odv-e66* | ODV envelopes | structural protein | |
| 108 | *ac75* | required for the nuclear egress of nucleocapsids and intranuclear microvesicle formation | structural protein | |
| 15 | *p49* | affect DNA synthesis | structural protein | |
| 92 | *p18* | core gene, egress of nucleocapsids | structural protein | |
| 138 | *vp1054* | capsid protein | structural protein | |
| 106 |  | structural protein in ODV and BV | structural protein | |
| 8 | ac146 | BV production | structural protein | |
| 71 | *p24capsid* | ODV component and associated with BV | structural protein | |
| 1 | *granulin* | structural protein | structural protein | |
| 75 |  | per os infectivity factors | structural protein | |
| 3 | *pk1* | catalytic domain activity | | structural protein |
| 97 | *odv-ec27* | ODV and BV associated and affect nucleocapsid formation | | structural protein |
| 104 | *gp41* | located between the virion envelope and capsid | | structural protein |
| 60 | *p74* | first identified per os infectivity factor | | structural protein |
| 85 | *bv/odv-c42 (p40)* | encodes a capsid-associated protein of BV and ODV | | structural protein |
| 57 | *pp31/39K* | capable of binding to DNA but not a virion structural protein | | virus transcription |
| 68 | *p47* | transient late gene expression and component of late gene RNA polymerase complex | | virus transcription |
| 95 | *lef-4* | component of the late baculovirus RNA polymerase | | virus transcription |
| 131 | *lef-8* | baculovirus RNA polymerase subunit | | virus transcription |
| 117 | *lef-9* | baculovirus RNA polymerase subunit | | virus transcription |
| 7 | *ie-1* | transient DNA replication | | virus transcription |
| 26 |  |  | |  |
| 49 |  |  | |  |
| 61 |  |  | |  |
| 27 |  |  | |  |
| 6 |  |  | |  |
| 36b |  |  | |  |
| 62 |  |  | |  |
| 122 |  |  | |  |
| 39 |  |  | |  |
| 70 |  |  | |  |
| 79 |  |  | |  |
| 25 |  |  | |  |
| 36a |  |  | |  |
| 32 |  |  | |  |
| 34 |  |  | |  |
| 72 |  |  | |  |
| 109 |  |  | |  |
| 121 |  |  | |  |
| 82a |  |  | |  |
| 45 |  |  | |  |
| 12 |  |  | |  |
| 20 | orf16L |  | |  |
| 129/130 |  |  | |  |
| 42 | orf35a |  | |  |
| 105 | ac78 |  | |  |
| 2 |  |  | |  |
| 50/51 |  |  | |  |
| 77 |  |  | |  |
| 139 |  |  | |  |
| 100 |  |  | |  |
| 43 |  |  | |  |
| 135 |  |  | |  |
| 110 |  |  | |  |
| 132 |  |  | |  |
| 33 |  |  | |  |
| 119 |  |  | |  |
| 115 |  |  | |  |
| 103 | ac81 |  | |  |
| 16 |  |  | |  |
| 44 | orf36L |  | |  |
| 52a |  |  | |  |
| 99 |  |  | |  |
| 28/29 |  |  | |  |

| **Supplementary Table S3.** isolate and genome group specific SNPs. Genome group A specific SNP was represented by all combined genome group but did not appear in group A. Different genome specific group A (CpGV-M), B (CpGV-E2), D (CpGV-I12), E (CpGV-S and -WW), F (CpGV-ZY2 and -JQ) and G (CpGV-ALE) were obtained from (Fan et al., 2020; Wennmann et al., 2017). Genome specific group A* indicated that SNP positions that were variable for all isolates except CpGV-M, were counted as CpGV-M specific. | | |
| --- | --- | --- |
| **Isolate group** | **Group count** | **Genome specific group** |
| ZY2_JQ | 89 | F |
| WW_ZY2_KS2_KS1_ALE_JQ_ZY_S_I12 | 75 | DEFG |
| E2 | 68 | B |
| WW_ZY2_KS2_KS1_ALE_JQ_ZY_S_I12_I0X_E2 | 58 | A* |
| WW_ZY2_KS2_KS1_ALE_JQ_ZY_S_I12_E2 | 45 | BDEFG |
| ZY2 | 30 | F |
| I12_I0X | 24 | D |
| ALE_JQ | 22 | FG |
| ALE | 21 | G |
| WW_ZY2_KS2_KS1_ALE_JQ_ZY_S_M_I12_I0X_E2 | 19 | ABDEFG |
| ZY2_JQ_I12_I0X | 17 | DF |
| I0X | 16 |  |
| S_I12 | 15 | DE |
| WW_ZY2_KS2_KS1_ALE_JQ_ZY | 12 | EFG |
| ZY2_JQ_E2 | 12 | BF |
| ALE_I12_I0X_E2 | 9 | BDG |
| JQ_I12_I0X | 9 | DF |
| ZY2_JQ_I12_I0X_E2 | 9 | BDF |
| I12 | 8 | D |
| JQ_I12_I0X_E2 | 6 | BDF |
| ZY2_I12_I0X | 6 | DF |
| ALE_JQ_I12_I0X | 5 | DFG |
| ALE_JQ_ZY | 5 | FG |
| JQ_I0X | 5 | F |
| WW_ZY2_KS2_KS1_ALE_JQ_ZY_S_I12_I0X | 5 | DEFG |
| WW_ZY2_KS2_KS1_ALE_JQ_ZY_S_M_I12_I0X | 5 | ADEFG |
| ZY2_JQ_S_I12_I0X_E2 | 5 | BDCEF |
| JQ | 4 | F |
| S_I12_E2 | 4 | BDE |
| WW_ZY2_KS2_KS1_ALE_JQ_ZY_S_M_I12_E2 | 4 | ABDEFG |
| ZY2_ALE_JQ | 4 | FG |
| ZY2_JQ_ZY | 4 | F |
| ALE_JQ_E2 | 3 | BFG |
| I12_I0X_E2 | 3 | BD |
| ZY2_JQ_I0X | 3 | F |
| ZY2_JQ_ZY_S_I12_I0X_E2 | 3 |  |
| ALE_JQ_M | 2 | AFG |
| KS1 | 2 | - |
| KS1_ZY_M_I12 | 2 | AD |
| KS2 | 2 | - |
| KS2_KS1_ALE_JQ_ZY | 2 | FG |
| M_I12_I0X_E2 | 2 | ABD |
| S_I12_I0X_E2 | 2 | BDE |
| WW_ZY2_JQ | 2 |  |
| WW_ZY2_KS2_KS1_ALE_JQ_ZY_E2 | 2 |  |
| WW_ZY2_KS2_KS1_JQ_ZY_S_I12_I0X_E2 | 2 | BDEF |
| ZY2_JQ_M_E2 | 2 |  |
| ZY2_ZY_I12_I0X | 2 |  |
| ALE_I0X | 1 | G |
| ALE_I12 | 1 | DG |
| ALE_I12_I0X | 1 | DG |
| ALE_JQ_I0X | 1 | FG |
| ALE_JQ_I12_I0X_E2 | 1 | BDFG |
| ALE_JQ_ZY_E2 | 1 | BFG |
| ALE_JQ_ZY_I12_I0X | 1 | DFG |
| ALE_JQ_ZY_I12_I0X_E2 | 1 | BDFG |
| JQ_S_I12 | 1 | DEF |
| JQ_S_I12_I0X | 1 | DEF |
| JQ_ZY_I12_I0X_E2 | 1 | BDF |
| JQ_ZY_S_M_I12 | 1 | ADEF |
| KS1_E2 | 1 | B |
| KS1_I12 | 1 | D |
| KS1_ZY | 1 | - |
| KS1_ZY_M | 1 | A |
| KS2_ALE_ZY_M_I12_I0X | 1 | ADG |
| KS2_I12 | 1 | D |
| KS2_I12_I0X | 1 | D |
| KS2_KS1_ALE_ZY_M_I12 | 1 | AD |
| KS2_KS1_ALE_ZY_M_I12_I0X | 1 | ADG |
| KS2_KS1_ZY | 1 | - |
| KS2_ZY_I12 | 1 | D |
| M | 1 | A |
| M_I12 | 1 | AD |
| M_I12_I0X | 1 | AD |
| S | 1 | E |
| S_I12_I0X | 1 | DE |
| WW_KS1_ALE_JQ_ZY | 1 |  |
| WW_KS1_S | 1 |  |
| WW_KS2_ALE_E2 | 1 |  |
| WW_KS2_KS1_ALE_JQ_ZY | 1 |  |
| WW_KS2_KS1_JQ_ZY_S_I12 | 1 |  |
| WW_ZY_S_I12_E2 | 1 |  |
| WW_ZY2_JQ_I12_I0X | 1 |  |
| WW_ZY2_JQ_ZY | 1 |  |
| WW_ZY2_KS1_JQ_S_I12_I0X_E2 | 1 |  |
| WW_ZY2_KS1_JQ_ZY_S_I12_E2 | 1 |  |
| WW_ZY2_KS2_KS1_ALE_JQ_S_I12_E2 | 1 |  |
| WW_ZY2_KS2_KS1_ALE_JQ_ZY_S_M_I12 | 1 | ADEFG |
| WW_ZY2_KS2_KS1_E2 | 1 |  |
| WW_ZY2_KS2_KS1_JQ_I12_E2 | 1 |  |
| WW_ZY2_KS2_KS1_JQ_I12_I0X_E2 | 1 |  |
| WW_ZY2_KS2_KS1_JQ_ZY_S_I12 | 1 | DEF |
| WW_ZY2_KS2_KS1_JQ_ZY_S_I12_E2 | 1 | BDEF |
| ZY_I12 | 1 |  |
| ZY_M | 1 |  |
| ZY_S_I12_I0X_E2 | 1 |  |
| ZY2_ALE_JQ_I12_I0X | 1 |  |
| ZY2_ALE_JQ_I12_I0X_E2 | 1 |  |
| ZY2_ALE_JQ_S_I12_I0X_E2 | 1 |  |
| ZY2_JQ_S_I12 | 1 | DEF |
| ZY2_JQ_S_I12_E2 | 1 | BDEF |
| ZY2_JQ_S_I12_I0X | 1 |  |
| ZY2_JQ_S_M_I12_I0X_E2 | 1 |  |
| ZY2_JQ_ZY_I12_I0X | 1 |  |
| ZY2_JQ_ZY_S_I12 | 1 |  |
| ZY2_JQ_ZY_S_I12_I0X | 1 |  |
| ZY2_KS1_ALE_JQ | 1 |  |
| ZY2_KS1_ALE_ZY_S_I12_I0X_E2 | 1 |  |
| ZY2_KS1_JQ_ZY_S_I12_E2 | 1 |  |
| ZY2_KS1_ZY_M | 1 |  |
| ZY2_KS2_JQ_ZY_S_I12_I0X_E2 | 1 |  |
| ZY2_KS2_KS1_ALE_ZY_M_I12_I0X | 1 | ADFG |
| ZY2_M_I12_I0X | 1 |  |
| ZY2_S_I12_E2 | 1 |  |
| ZY2_ZY_M_I12_I0X_E2 | 1 |  |

References

[As per the main references, you may want to use Rohrmann’s most recent 4^th^ Edition, but you may need to double check to make sure the 4^th^ edition is not different from the 3^rd^ in your gene annotations]

Bergin, D., Reeves, E.P., Renwick, J., Wientjes, F.B., Kavanagh, K., 2005. Superoxide Production in Galleria mellonella Hemocytes: Identification of Proteins Homologous to the NADPH Oxidase Complex of Human Neutrophils. Infection and Immunity 73, 4161-4170.

Biswas, S., Willis, L.G., Fang, M., Nie, Y., A., T.D., 2018. Autographa californica Nucleopolyhedrovirus AC141 (Exon0), a Potential E3 Ubiquitin Ligase, Interacts with Viral Ubiquitin and AC66 To Facilitate Nucleocapsid Egress. J Virol 92, e01713-01717.

Braunagel, S.C., Russell, W.K., Rosas-Acosta, G., Russell, D.H., Summers, M.D., 2003. Determination of the protein composition of the occlusion-derived virus of Autographa californica nucleopolyhedrovirus. Proc Natl Acad Sci U S A 100, 9797-9802.

Fan, J., Wennmann, J.T., Wang, D., Jehle, J.A., 2020. Single nucleotide polymorphism (SNP) frequencies and distribution reveal complex genetic composition of seven novel natural isolates of Cydia pomonella granulovirus. Virology 541, 32-40.

Garavaglia, M.J., Miele, S.A., Iserte, J.A., Belaich, M.N., Ghiringhelli, P.D., 2012. The *ac53, ac78, ac101*, and *ac103* genes are newly discovered core genes in the family *Baculoviridae*. J Virol 86, 12069-12079.

Guarino, L.A., 1990. Identification of a viral gene encoding a ubiquitin-like protein. Proceedings of the National Academy of Sciences 87, 409-413.

Gueli Alletti, G., Sauer, A.J., Weihrauch, B., Fritsch, E., Undorf-Spahn, K., Wennmann, J.T., Jehle, J.A., 2017. Using next generation sequencing to identify and quantify the genetic composition of resistance-breaking commercial isolates of Cydia pomonella granulovirus. Viruses 9, 250.

Guo, Y.-J., Fu, S.-H., Li, L.-L., 2017. Autographa californica multiple nucleopolyhedrovirus ac75 is required for egress of nucleocapsids from the nucleus and formation of *de novo* intranuclear membrane microvesicles. PLoS One 12, e0185630-e0185630.

Han, Y., van Houte, S., van Oers, M., Ros, V., 2018. Baculovirus PTP2 Functions as a Pro-Apoptotic Protein. Viruses 10, 181.

Lai, Q., Wu, W., Li, A., Wang, W., Yuan, M., Yang, K., 2018. The 38K-Mediated Specific Dephosphorylation of the Viral Core Protein P6.9 Plays an Important Role in the Nucleocapsid Assembly of Autographa californica Multiple Nucleopolyhedrovirus. J Virol 92, e01989-01917.

Lapointe, R., Popham, H.J.R., Straschil, U., Goulding, D., O'Reilly, D.R., Olszewski, J.A., 2004. Characterization of Two Autographa californica Nucleopolyhedrovirus Proteins, Ac145 and Ac150, Which Affect Oral Infectivity in a Host-Dependent Manner. J Virol 78, 6439-6448.

Luque, T., Finch, R., Crook, N., O'Reilly, D.R., Winstanley, D., 2001. The complete sequence of the Cydia pomonella granulovirus genome. J Gen Virol 82, 2531-2547.

Nan, H., Chen, H., Tuite, M.F., Xu, X., 2019. A viral expression factor behaves as a prion. Nat Commun 10, 359.

Ono, C., Kamagata, T., Taka, H., Sahara, K., Asano, S.-i., Bando, H., 2012. Phenotypic grouping of 141 BmNPVs lacking viral gene sequences. Virus Res 165, 197-206.

Pietruska, D., 2018. Vergleichende Transkriptomanalysen des Cydia pomonella granulovirus (CpGV) in sensiblen und resistenten Apfelwicklern (*Cydia pomonella*). Julius Kühn-Institut, Bundesforschungsinstitut für Kulturpflanzen, Quedlinburg, Germany, p. 199.

Reilly, L.M., Guarino, L.A., 1996. The Viral Ubiquitin Gene of Autographa californicaNuclear Polyhedrosis Virus Is Not Essential for Viral Replication. Virology 218, 243-247.

Rohrmann, G.F., 2013. in: 3rd (Ed.), Baculovirus Molecular Biology. National Center for Biotechnology Information (US) George Rohrmann., Bethesda (MD).

Rosas-Acosta, G., Braunagel, S.C., Summers, M.D., 2001. Effects of Deletion and Overexpression of the Autographa californica Nuclear Polyhedrosis Virus FP25KGene on Synthesis of Two Occlusion-Derived Virus Envelope Proteins and Their Transport into Virus-Induced Intranuclear Membranes. J Virol 75, 10829-10842.

Tomalski, M.D., Eldridge, R., Miller, L.K., 1991. A baculovirus homolog of aCu/Znsuperoxide dismutase gene. Virology 184, 149-161.

Wang, D., Zhang, C.-X., 2006. HearSNPV *orf83* encodes a late, nonstructural protein with an active chitin-binding domain. Virus Res 117, 237-243.

Wei, D., Wang, Y., Zhang, X., Hu, Z., Yuan, M., Yang, K., 2014. Autographa californica Nucleopolyhedrovirus Ac76: a Dimeric Type II Integral Membrane Protein That Contains an Inner Nuclear Membrane-Sorting Motif. J Virol 88, 1090-1103.

Wennmann, J.T., Radtke, P., Eberle, K.E., Gueli Alletti, G., Jehle, J.A., 2017. Deciphering single nucleotide polymorphisms and evolutionary trends in isolates of the Cydia pomonella granulovirus. Viruses 9, 227.

Wilson, M.E., Mainprize, T.H., Friesen, P.D., Miller, L.K., 1987. Location, transcription, and sequence of a baculovirus gene encoding a small arginine-rich polypeptide. J Virol 61, 661-666.

Wu, W., Passarelli, A.L., 2012. The Autographa californica M Nucleopolyhedrovirus ac79 Gene Encodes an Early Gene Product with Structural Similarities to UvrC and Intron-Encoded Endonucleases That Is Required for Efficient Budded Virus Production. J Virol 86, 5614-5625.

Xu, X., Zhou, X., Nan, H., Zhao, Y., Bai, Y., Ou, Y., Chen, H., 2016. Aggregation of AcMNPV LEF-10 and Its Impact on Viral Late Gene Expression. PLoS One 11, e0154835.
